# To expand or not to expand?

**DOI:** 10.1590/2177-6709.25.1.007-008.edt

**Published:** 2020

**Authors:** Flavia Artese

**Affiliations:** 1 Universidade do Estado do Rio de Janeiro, Departamento de Odontologia Preventiva e Comunitária (Rio de Janeiro/RJ, Brazil).

During my orthodontic training, in the early 90’s, the lower arch dimensions were inviolable. On the other hand, expansions of the upper arch were obtained, in children, by palatal expansions, while in adults they were carried out by means of surgically-assisted expansion. However, not always the face and the malocclusion have the same needs. It isn’t rare to be in doubt on when to align teeth in patients with concave profiles, that would not benefit from tooth extractions. On the contrary, they could much benefit from dental expansion and tooth projection. 

This first 2020 edition of the *Dental Press Journal of Orthodontics* has a series of papers that truly contribute to a clinical dilemma: the need of obtaining space in the dental arches without extractions.

During the early 2000’s, with the introduction of self-ligated brackets, we watched a stimulus to significant arch expansions. At the time, it was proposed that the decreased friction between the archwire and the bracket would stimulate bone growth, with osseous neoformation in the expansion areas, so called arch development. Yet one of the studies published in this edition (pgs 47-55), performed by Spanish researchers, clearly demonstrates that there are no differences in the dimensions of arches expanded with self-ligated or conventional brackets. It is also clear that expansion occurs independent of the type of brackets used. 

Considering that we do perform arch expansion, be it with orthodontic archwires or with intraoral appliances, what do we know about these expansions? What are our limits? What should be the consequences of extrapolating the dentoalveolar envelope? The BBO case report (pgs 70-79) describes the treatment of a 14 year old girl with evident maxillary constriction, that used a Hyrax expander. In this treatment approach, it was observed that the midpalatal suture did not open and expansion was of a dentoalveolar nature. This paper discusses contemporary methods to evaluate suture maturation with CBCTs, and argues that the results in these cases are obtained by dentoalveolar changes. This type of expansion happens due to posterior teeth uprighting and a resulting bone remodeling in this direction.^1^ Nevertheless, long-term studies show that the incidence of gingival recession is greater in the upper molar and premolar regions^2^ in patients submitted to dentoalveolar expansion. They also show that expansions of 1 to 5 mm are an acceptable alternative to extractions, if there is a three-dimensional control of the movement,^3^ as well as good gingival health and appropriate phenotype. 

Factors that were associated to a smaller risk for gingival recession are keratinized gingival height, bone width and final dimension of intercanine width. For each mm in height of keratinized gingiva there was 80% less chance of recession. For each mm of bone width, there was 116% less chance of gingival recession, and for each mm of increase in lower intercanine width there was 39 to 47% greater chance of recession.^4^

The frequency of gingival recessions seems not to increase with orthodontic treatment in young patients.^5^ But it is known that it is more prevalent in the adult population, occurring in 38% of patients aged more than 30 years in at least one tooth, most commonly in the mid portion of the dental arch.^5^ Therefore, a judicious diagnosis of the risk factors for gingival recession should be performed when expanding in adults.

Two other papers in this edition approach this problem. The first one, by Figueiredo et al. (pgs 56-63), evaluated the esthetic impact of upper canines with recession and demonstrated that the presence of this situation can negatively impact smile esthetics. The Special Topic, written by Dr. Carlos Alexandre Camara (pgs 80-88), also presents a visual diagnostic tool, with the use of the SmileCurves Template, for upper canine inclinations. 

To expand or not to expand is a multi-factorial decision and goes way beyond our wishes or even the patients’ desires. The adequacy of dental volume over bone volume will always present limits that should be evaluated and considered in treatment planning. There are no magical appliances and we have not yet discovered non-invasive methods to create bone on the buccal surface of teeth. In reality, to solve our clinical dilemma, information is always very welcome, with the intention of performing a non-iatrogenic practice - and in this edition of the DPJO we offer you much knowledge to expand.

I invite and stimulate you to this reading.
